# Familial imbalance in 16p13.11 leads to a dosage compensation rearrangement in an unaffected carrier

**DOI:** 10.1186/s12881-014-0116-3

**Published:** 2014-10-29

**Authors:** Alicia Delicado, Luis Fernández, María Luisa de Torres, Julián Nevado, Fe Amalia García-Santiago, Roberto Rodríguez, Elena Mansilla, María Palomares, Fernando Santos-Simarro, Elena Vallespín, María Ángeles Mori, Pablo Lapunzina

**Affiliations:** Instituto de Genética Médica y Molecular (INGEMM), IdiPAZ, Hospital Universitario La Paz, Paseo de la Castellana 261, 28046 Madrid, Spain; CIBER de Enfermedades Raras (CIBERER), ISCIII, Madrid, Spain; Servicio de Fisiopatología Fetal, Hospital Universitario La Paz, Madrid, Spain

**Keywords:** 16p13.11 duplication, Gene dosage compensation, Homologous balanced rearrangement, Mitotic NAHR

## Abstract

**Background:**

We and others have previously reported that familial cytogenetic studies in apparently *de novo* genomic imbalances may reveal complex or uncommon inheritance mechanisms.

**Methods:**

A familial, combined genomic and cytogenetic approach was systematically applied to the parents of all patients with unbalanced genome copy number changes.

**Results:**

Discordant array-CGH and FISH results in the mother of a child with a prenatally detected 16p13.11 interstitial microduplication disclosed a balanced uncommon rearrangement in this chromosomal region. Further dosage and haplotype familial studies revealed that both the maternal grandfather and uncle had also the same 16p duplication as the proband. Genomic compensation observed in the mother probably occurred as a consequence of interchromosomal postzygotic nonallelic homologous recombination.

**Conclusions:**

We emphasize that such a dualistic strategy is essential for the full characterization of genomic rearrangements as well as for appropriate genetic counseling.

## Background

The combination of array-based comparative genomic hybridization (aCGH) technology and subsequent parental fluorescence *in situ* hybridization (FISH) analysis looking for balanced parental rearrangements has revealed complex or unusual genomic rearrangements that seem more common than previously considered [1-3]. Recently, it has been also demonstrated that parental submicroscopic insertional translocations (ITs) underlie ~2.1% of the apparently *de novo* interstitial pathogenic copy number variations (CNVs) [4].

At the same time, the clinical use of aCGH has lead to the detection of many CNVs, a number of them of uncertain clinical relevance. Patients with 16p13.11 duplication and its reciprocal deletion have been previously reported and are relatively common. However, whereas the 16p13.11 deletion showed to be a pathogenic CNV, the duplication was initially thought to be benign [5]. Other authors described an incomplete penetrance and/or variable expressivity, the same duplication being found both in mildly affected and unaffected relatives within the same family [6,7]. Clinical features such as cognitive impairment, behavioral disorders, congenital heart defects, skeletal malformations and abnormal magnetic resonance imaging (MRI) findings have been associated with CNVs involving 16p13.11 [8].

In this report we describe a child with a brain malformation detected by prenatal ultrasound in which a prenatal aCGH study identified a 1.78 Mb duplication at 16p13.11. Further parental FISH analysis showed that the phenotypically normal mother was carrying an unusual apparently balanced rearrangement between the two chromosome 16 homologs. An elder brother of the patient, and both the patient’s maternal grandfather and maternal uncle, all three apparently phenotypically normal, were also carriers of the same 16p duplication detected in the proband.

## Methods

### Clinical report

Samples from the proband and his family were obtained after informed consent. Ethical approval was obtained for this study from the IRB at Hospital Universitario La Paz in Madrid (HULP-CEIC-PI1207). Research was performed in compliance with the Declaration of Helsinki.

The patient, a male, was the product of the third pregnancy of a 34 year old woman. Both parents were healthy, phenotypically normal and nonconsanguineous. The first pregnancy ended in an elective termination and the second resulted in a healthy boy. A prenatal ultrasound at 28 weeks gestation revealed agenesis of corpus callosum. Further examination at 37 weeks showed two interhemispheric cysts along with corpus callosum agenesis and colpocephaly. He was born at 40 weeks of gestation by spontaneous vaginal delivery. At birth, weight was 3660 g (75–90th centile), length 52 cm (90th centile) and head circumference 36 cm (90th centile). Perinatal period was uneventful and Apgar scores were 9 and 10 at 5 and 10 minutes, respectively. Renal ultrasound showed mild bilateral pelvicalyceal dilatation.

Although initial marsupialisation of the cysts carried out at 20 days of life showed some reduction in their volume, subsequent increase in their size despite multiple drainage interventions mandated the placement of a permanent ventriculoperitoneal shunt at 10 months of age.

He was last seen in clinic aged 14 months, the brain MRI showing a 50% reduction in cysts volume. At that time, his developmental evaluation disclosed a normal cognitive profile for age with a motor delay secondary to left side hemiparesis.

His older brother was assessed in clinic aged 3, showing at that time a normal phenotype, normal growth parameters and a normal development for his age. The maternal uncle and grandparents were not directly evaluated in the genetics clinic, but were referred as normal by other family members.

### Genomic and cytogenetic studies

According to our workflow in prenatal samples with two or more ecographic markers, aCGH was performed in the patient and his parents, using a custom oligonucleotide array (KaryoArray® v3.0, 8 × 60 K, Agilent Technologies, Santa Clara, CA) as described previously [9]. This array has an average density of one probe per 9 Kb in clinically relevant regions (microdeletion/microduplication syndromes, subtelomeric and pericentromeric regions) and one probe per 175 Kb in other genomic regions including 16p13.11 (backbone). Further versions of this array will include this and other regions as they are associated with clinical phenotypes.

Karyotyping and FISH were performed on cultured peripheral blood lymphocytes. FISH studies were performed using standard procedures applying probe NDE1-MYH11 mapping 16p13.11 (Agilent Technologies, Inc., Santa Clara, CA).

Short tandem repeat (STR) markers segregation studies were carried out in the family to confirm gene dosage and to determine the parental origin of the rearranged chromosomal material. Genomic DNA was obtained using standard procedures from peripheral blood lymphocytes in all family members (QIAGEN, Valencia, CA), and from buccal swab in the mother (DNA Genotek, Kanata, Canada). Three polymorphic markers mapping to region 16p13.11 (D16S3060: 15,860,105-15,860,300; D16S3127: 15,870,885-15871004; D16S405: 15,882,592-15,882,733; hg19) and other nine markers located along chromosome 16 (D16S3070, D16S3088, D16S3024, D16S3079, D16S3114, D16S500, D16S690, D16S753, D16S3043) were analyzed.

Further studies to determine the presence of the gene dosage alteration in other family members were also carried out by Multiplex Ligation-dependent Probe Amplification (MLPA) using SALSA P092-B3 probemix containing probes for genes *ABCC6* and *ABCC1* in 16p13.11 (MRC-Holland, Amsterdam, The Netherlands).

## Results

**Figure 1:**
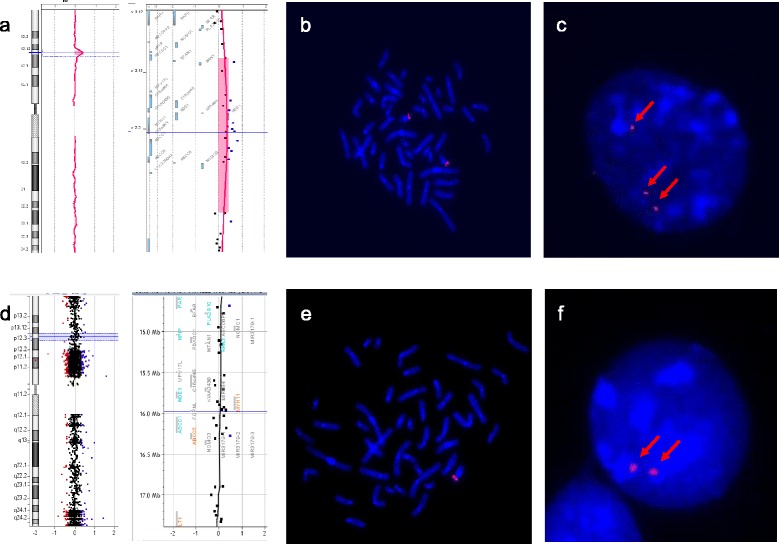
**aCGH and FISH results in the proband and the mother. a)** Proband aCGH results showing 16p13.11 duplication. **b-c)** Proband FISH results with probe NDE1-MYH11 (red) showing two signals on the metaphase chromosomes **(b)** and three signals on the interphase nucleus **(c)**. **d)** Mother aCGH results showing no 16p13.11 duplication. **e-f)** Mother FISH results with the same probe showing one signal on metaphase chromosomes **(e)** and two signals on the interphase nucleus **(f)**.

Array comparative genomic hybridization in the patient revealed a gain of 1.78 Mb on 16p12.3-p13.11 (hg19, chromosome 16: 15,111,247-16,895,894) (Figure 1a). Genes included in this region are: *PDXDC1*, *NTAN1*, *RRN3*, *MPV17L*, *C16orf45*, *KIAA0430*, *MIR484*, *NDE1* (MIM 609449), *MYH11* (MIM 160745), *C16orf63*, *ABCC1*, *ABCC6* (MIM 603234), *NOMO3*, *PKD1P1*. This finding was interpreted as a CNV of uncertain significance since this region had been reported to show copy number variation in individuals with no obvious phenotype, it contained several MIM genes and there was an ongoing debate as to the exact clinical significance of the CNV. Further aCGH studies on both parents were normal (Figure 1d) thus the duplication was initially considered an apparently *de novo* event in the boy.

Duplication in the proband could have hardly been detected in metaphase FISH studies, although retrospective re-evaluation of images noted a brighter signal pattern on one homologue (Figure 1b). Further interphase nuclei analysis with the same probe demonstrated actually three signals, two of them located in close proximity (Figure 1c). These findings supported the results of aCGH and allowed us to establish that the gene dosage amplification observed in the patient was located within that same region. His karyotype was then described as: ish dup(16)(p12.3p13.11)(NDE1-MYH11enh). nuc ish 16p13.11(NDE1-MYH11x3).

Discordant to the aCGH result, metaphase FISH analysis in the mother showed a deletion pattern, with only one probe signal on one chromosome 16. This signal was also brighter than usual, suggesting that two copies of 16p13.11 were present on that chromosome (Figure 1e). Further interphase nuclei FISH testing was then performed, and two signals adjacent to one another were observed (Figure 1f). Therefore, in combination with the aCGH result, FISH findings were interpreted as a balanced interchromosomal rearrangement at 16p12.3-p13.11, resulting in one chromosome 16 with an interstitial deletion and the other with an interstitial insertion. The karyotype of the mother was described as follows: ish der(16)ins(16)(16;16)(p1?2.3;p1?2.3p1?3.11)(NDE1-MYH11enh),der(16)ins(16)(16;16)(p1?2.3;p1?2.3p1?3.11)(NDE1-MYH11-). nuc ish 16p13.11(NDE1-MYH11x2).

Interpretation of the rearrangement in the mother and its associated recurrence risk focused attention on the proband’s elder brother. By means of MLPA, the same results as in the proband were obtained, indicating that both children had inherited the derivative maternal chromosome 16 carrying two copies of 16p13.11. Thus, their karyotypes were described as: ish der(16)ins(16)(16;16)(p1?2.3;p1?2.3p1?3.11)(NDE1-MYH11enh)mat.

Surprisingly, further familial studies showed that both the maternal grandfather and the maternal uncle also carried the same duplication as the proband.

**Figure 2:**
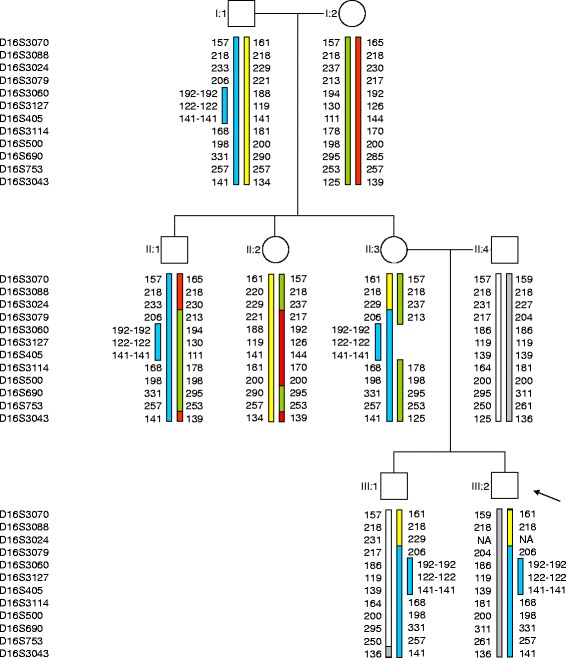
**Pedigree chart showing haplotypes of 12 markers spanning chromosome 16.** Markers D16S3060, D16S3127 and D16S405 are located within the rearranged segment 16p13.11, which shows extra dosage in individuals I:1, II:1, II:3, III:1 and III:2. Colors indicate the grandparental origin of the chromosomal material. Numbers designate the size in bp of the fragment including the marker.

The analysis of three STR markers mapping within the region delimited by aCGH supported previous gene dosage results in the children, maternal grandfather, and maternal uncle. In the mother, the grandpaternal genotype was observed in two of the three markers. However, all other informative markers (6) in chromosome 16 showed biparental inheritance. The same results were obtained in DNA from buccal swab in the mother, and they are consistent with a deletion of the maternal allele of 16p13.11 (Figure 2).

## Discussion

Only a few cases describing homologous balanced rearrangements have been reported so far. Most of them occurred at 22q11.2 region [1-3], which is well known for being rich in low copy repeats (LCRs). Most constitutional translocations involving 22q11 share the same 22q11.2 breakpoint located within LCR-B, and these breakpoints are usually located at the center of palindromic AT-rich repeat sequences (PATRRs) [10]. Therefore, palindrome-mediated translocations have been suggested as one of the mechanisms for human chromosomal rearrangements. The short arm of chromosome 16 has a similar structure with over 10% of the euchromatic region being composed of LCRs which promote non-allelic homologous recombination (NAHR) [7,11,12].

**Figure 3:**
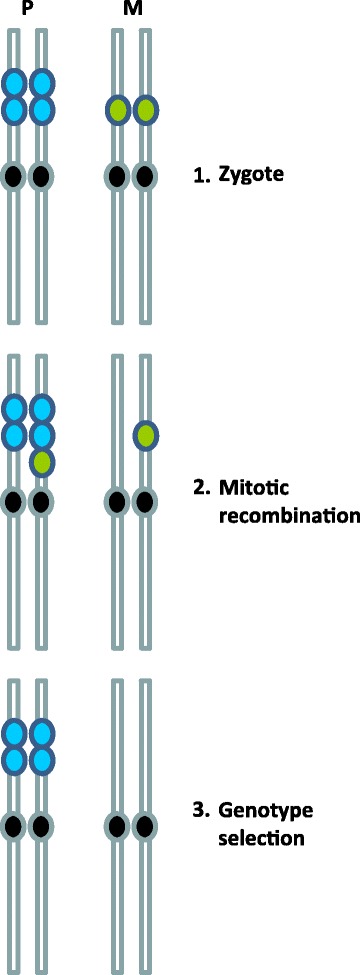
**Postzygotic hypothesis for the rearrangement in chromosome 16 leading to genomic compensation in the mother (II:3).** Mitotic recombination between a paternal chromatid carrying the duplication and a normal maternal chromatid during the first zygotic divisions would originate four different chromatids, two of which would be selected in a compensated genotype. Circles indicate region 16p13.11, which is colored as in Figure 2. P and M designate the paternal and maternal chromosome, respectively.

Haplotype analysis suggests two possible mechanisms underlying the origin of the balanced rearrangement in the proband’s mother. We can hypothesize that previous to conception, a meiotic NAHR might have occurred in the proband’s grandmother (we also cannot exclude the possibility of a germline mosaicism) with the subsequent transmission to the proband’s mother of the deleted chromosome 16. A duplicated chromosome 16 would have been transmitted by the proband’s grandfather. A second and more likely mechanism is a mitotic NAHR between a maternal and paternal chromatid within the first zygotic divisions that would originate four chromatids: the two recombination products (one with a triplication and the other one with the reciprocal deletion) and two original parental chromatids, carrying a duplication and a normal dosage. This event would be followed by selection of cells carrying the two compensated non-sister chromatids in the embryo, in a model similar to the one proposed by Carelle-Calmels *et al.* [1] (Figure 3).

A consequence of postzygotic NAHR is chromosomal mosaicism. Studies in the mother revealed the same genotype in buccal swab and peripheral blood lymphocytes, suggesting that cell lineages with different genotypes might have been restricted to other tissues or would have been confined to extraembryonic tissues, a likely explanation if the NAHR occurred in fact early after conception.

LCR-mediated NAHR may originate unstable products that are more prone to act as substrates of new rearrangements in further generations [4]. In this case, it is noteworthy that, considering the meiotic recombinations observed in the grandparental gametes, both the proband’s mother and maternal uncle received the same parental genotypic contribution in 16p13.11, but the proposed rearrangement occurred only in the mother (Figure 2). This rearrangement resulted in gene dosage compensation, preventing the possible phenotypic effects of the CNV in that woman.

The phenotype of patients with 16p13.11 duplication is very variable and it has been associated with clinical features including behavioral abnormalities, autistic spectrum disorders, congenital heart defects, skeletal manifestations, and developmental delay [8]. Significant association has been described for schizophrenia and intellectual disability [13], and brain malformations have been sporadically observed [14]. Psychomotor development in the proband and his brother was normal at the time of the assessment, although future cognitive handicap cannot be ruled out. *NDE1*, one of the genes included in the duplicated segment, is strongly expressed in brain, and it forms complexes with *LIS1*, a dosage-sensitive gene crucial for neuronal migration and cerebral development [15] and known to underlie Miller–Dieker lissencephaly syndrome (MIM 247200) [5]. Notwithstanding, since the CNV is also present in other healthy family members, we cannot associate it to the cerebral malformations observed in the proband so it still remains as a finding of uncertain significance.

On the other hand, this uncommon rearrangement in chromosome 16 in the mother of the proband raises the risk of an unbalanced pregnancy to 100%, since all of her offspring will either inherit the 16p13.11 duplication or the deletion. The detection of this unusual rearrangement emphasizes the need of parental FISH studies in order to be able to offer accurate genetic counseling for future pregnancies.

## Conclusions

The increasing detection of these unusual rearrangements reinforces the need to determine the genomic location of interstitial gains or losses detected by aCGH [16]. Consequently, combined *in situ* hybridization and genomic parental approaches are crucial in order to rule out any balanced parental rearrangement that may involve a very high recurrence risk.

## Abbreviations

aCGH: Array-based comparative genomic hybridization
CNV: Copy number variation
FISH: Fluorescence *in situ* hybridization
IT: Insertional translocation
LCR: Low copy repeat
MLPA: Multiplex ligation-dependent probe amplification
MRI: Magnetic resonance imaging
NAHR: Non-allelic homologous recombination
PATRR: Palindromic AT-rich repeat
STR: Short tandem repeat
